# Extracorporeal cardiopulmonary resuscitation for refractory cardiac arrest: an overview of current practice and evidence

**DOI:** 10.1007/s12471-023-01853-5

**Published:** 2024-02-20

**Authors:** Samir Ali, Christiaan L. Meuwese, Xavier J. R. Moors, Dirk W. Donker, Anina F. van de Koolwijk, Marcel C. G. van de Poll, Diederik Gommers, Dinis Dos Reis Miranda

**Affiliations:** 1 Department of Intensive Care, Erasmus University Medical Centre, Rotterdam, The Netherlands; 2 Department of Anaesthesiology, Erasmus Medical Centre, Rotterdam, The Netherlands; 3 Ministry of Defence, Royal Netherlands Air Force, Breda, The Netherlands; 4 Department of Cardiology, Erasmus University Medical Centre, Rotterdam, The Netherlands; 5 Helicopter Emergency Medical Services, Trauma Centre Zuid-West Nederland, Erasmus University Medical Centre, Rotterdam, The Netherlands; 6 Cardiovascular and Respiratory Physiology, Faculty of Science and Technology, University of Twente, Enschede, The Netherlands; 7 Department of Intensive Care, University Medical Centre Utrecht, Utrecht, The Netherlands; 8 Department of Intensive Care, Maastricht University Medical Centre, Maastricht, The Netherlands

**Keywords:** Out-of-hospital cardiac arrest, Extracorporeal membrane oxygenation, Cardiopulmonary resuscitation, Advanced cardiac life support

## Abstract

Cardiac arrest (CA) is a common and potentially avoidable cause of death, while constituting a substantial public health burden. Although survival rates for out-of-hospital cardiac arrest (OHCA) have improved in recent decades, the prognosis for refractory OHCA remains poor. The use of veno-arterial extracorporeal membrane oxygenation during cardiopulmonary resuscitation (ECPR) is increasingly being considered to support rescue measures when conventional cardiopulmonary resuscitation (CPR) fails. ECPR enables immediate haemodynamic and respiratory stabilisation of patients with CA who are refractory to conventional CPR and thereby reduces the low-flow time, promoting favourable neurological outcomes. In the case of refractory OHCA, multiple studies have shown beneficial effects in specific patient categories. However, ECPR might be more effective if it is implemented in the pre-hospital setting to reduce the low-flow time, thereby limiting permanent brain damage. The ongoing ON-SCENE trial might provide a definitive answer regarding the effectiveness of ECPR. The aim of this narrative review is to present the most recent literature available on ECPR and its current developments.

## Introduction

Out-of-hospital cardiac arrest (OHCA) occurs in 37 per 100,000 Dutch inhabitants [1] and is associated with a survival to hospital discharge rate of only 23% [1]. This survival rate has improved considerably in recent years and is currently among the highest in Europe [2]. Key factors contributing to this relatively good survival rate are basic life-support training of lay persons, introduction of a text message alert system and the availability of automated external defibrillators in public spaces [3].

Of all OHCA survivors, 90% have return of spontaneous circulation (ROSC) within 15 min of the collapse. However, when resuscitation time exceeds these 15 min, the proportion of patients with a favourable neurological outcome drops tremendously to below 10% [4]. This is the case in nearly 17% of patients with ventricular fibrillation [5] and often reflects a higher severity of the underlying (coronary artery) disease [6].

**Fig. 1:**
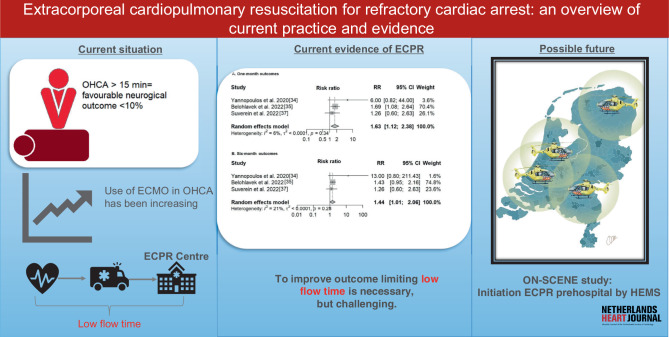
Infographic: Current practice and evidence

A promising intervention for patients with refractory OHCA is veno-arterial extracorporeal membrane oxygenation (V‑A ECMO) during cardiopulmonary resuscitation (ECPR) [7]. This review summarises current practice and evidence of ECPR in the setting of OHCA (Fig. 1: Infographic).

## Cardiac arrest and its detrimental effects

Cardiac arrest leads to a fatal outcome when a critical threshold of organ and tissue ischaemia is surpassed and circulation is not restored in due time. The rapid decay is initiated by circulatory collapse, which in turn triggers a cascade of pathophysiological events that inevitably result in irreversible organ damage. And while the initial injury is initiated by ischaemia, a second phase of injury starts after reperfusion [8] and is mediated by a systemic inflammatory response that constitutes part of the so-called post-cardiac arrest syndrome. The majority of organs have the capacity to regenerate or can be (partially) replaced, but severe brain damage is often irreversible and therefore of vital importance for quality of life and survival.

The primary injury, caused by hypoperfusion, has a detrimental effect on the blood-brain barrier and neurons by increasing vascular permeability, which will eventually lead to severe cellular dysfunction and cerebral oedema [9]. This already significant damage is aggravated by the effects of reperfusion, further disrupting calcium homeostasis, mitochondrial function, and inducing free radical formation and inflammation. These effects all contribute to an increase in cerebral oedema and direct cellular toxicity, eventually causing apoptosis [8].

For a good neurological outcome, it is pivotal to limit the no-flow and low-flow time, thereby mitigating the devastating injury to the brain. As such, it is essential to minimise time to reperfusion. Application of ECMO during cardiopulmonary resuscitation (CPR) could potentially restore and provide life-saving blood flow to the brain while the heart is still ineffective and, as such, has emerged as a promising support modality in this setting.

## The concept of ECPR

An ECMO machine is a mechanical device that can be used for refractory circulatory and/or respiratory failure [10]. The main mechanism of action is to actively drain blood from the central venous compartment, oxygenating and decarboxylating it in an oxygenator. The vascular compartment into which the blood is returned determines the type of support. When the return cannula is inserted in the venous compartment, only gas exchange occurs. If, however, blood is returned into the arterial system, the (malfunctioning) heart is bypassed too, providing circulatory support in addition to gas exchange.

Increasing experience with ECMO, as well as the broadening of indications for respiratory and circulatory support, resulted in a fast increase in ECMO applications [11, 12]. More recently, interest in the use of VA-ECMO in patients with refractory OHCA has rapidly increased. In this setting, ECMO support is established during the course of ongoing cardiac arrest and CPR. Vascular access is usually obtained by cannulating the femoral artery and femoral vein with 19–25 and 17–19 gauge cannulas, respectively. In the majority of instances, a percutaneous approach is utilised with ultrasound-guided puncturing of the vessel and the Seldinger technique. Surgical techniques have been described as an alternative approach for vascular access. Both approaches have their advantages and disadvantages ([13, 14]; Tab. 1). While vascular complications occur more commonly during a percutaneous approach [13], their incidence is low when cannulation is performed by experienced hands. This concept also holds for the pre-hospital situation. The degree of potential support largely depends on cannula size. After successful cannulation, VA-ECMO can immediately restore systemic blood flow and blood pressure, particularly limiting cerebral ischaemia, which provides time to identify and treat the underlying aetiology of the cardiac arrest.

**Table 1:** Advantages and disadvantages of percutaneous versus surgical vascular access

Surgical cannulation	Percutaneous cannulation
*Disadvantage*	
Higher number of bleeding complications	Higher number of vascular complications requiring surgery after decannulation [14]
Higher number of infection complications	
Higher in-hospital mortality	
*Advantage*	
Larger cannula	More readily available
Malplacement less probable (AA or VV cannulation)	

*AA* arterio-arterial, *VV* veno-venous

For limiting the devastating effects of the post-cardiac-arrest syndrome, it has been advocated that patients could benefit from an initially high ECMO flow and special composition of the priming solution and arterialised blood [15]. Observations supporting this hypothesis [16] have led to the development of a dedicated ECMO console called ‘Controlled Automated Reperfusion of the whoLe body (CARL)’, which was designed to limit the detrimental effects of reperfusion. For this purpose, a modified VA-ECMO device is utilised which can provide pulsatile flow and performs several additional functions, including the ability to lower body temperature, significantly decrease serum calcium levels thus preventing intracellular calcium influx, maintain serum pH and osmolality within certain ranges to lower metabolic activity and prevent cerebral oedema. Initial results from a pilot study on CARL seem promising, but more research is needed [15].

## Indications for ECPR

For refractory OHCA with a presumed cardiac origin, most Dutch ECMO centres consider the application of ECPR in patients below 70 years of age, with ventricular fibrillation/ventricular tachycardia as the first observed rhythm, and initiation of bystander basic life support. In addition, the expected time from collapse to start of cannulation should not exceed 60 min (Tab. 2). For non-cardiac causes of refractory OHCA, ECPR initiation protocols differ largely between different Dutch ECMO centres. Patients with OHCA due to intoxications primarily affecting the circulation, near-drowning, hypothermia and massive pulmonary embolism are considered to be candidates in some Dutch ECMO centres, while they are not found to be eligible in other centres [17]. This treatment variation is also observed in the international literature [18–21]. For patients with suspected pulmonary embolism, ECMO can be used in the first phase to stabilise and regain circulation, followed by a (percutaneous) thrombectomy [22]. In such instance, evidence suggests that combining surgical thrombectomy with VA-ECMO is more effective than using VA-ECMO alone or along with thrombolysis [23]. It should, however, be noted that such evidence was typically obtained in patients in whom thrombolysis failed. Up to now, thrombolysis remains the treatment of first choice for massive pulmonary embolism [24].

**Table 2:** Criteria during cardiac arrest associated with favourable and unfavourable outcomes

Favourable criteria	Unfavourable criteria
Age < 70 years	Last seen well > 5 min
Witnessed cardiac arrest	No flow > 5 min
Initial shockable rhythm	Non-shockable rhythm
EtCO_2_ > 10 mm Hg	
Signs of life during CPR	
Expected time to cannulation after CA < 60 min	

*EtCO*_*2*_ end-tidal CO2 levels, *CPR* cardiopulmonary resuscitation, *CA* cardiac arrest

## Evidence regarding the effects of ECPR

The European [25] and American [26] guidelines all recommend considering ECPR in the case of refractory OHCA. In the OHCA setting, discrepant findings have been reported. Yannopoulos et al. found that the incidence of in-hospital survival with favourable neurological recovery (Cerebral Performance Category [CPC] scores 1–2) was 27% percent higher for ECPR patients versus historical controls [6]. The CPC is a recommended five-point scale utilised for classifying neurological outcomes following cardiac arrest [27]. Another observational study, however, showed no effect of ECPR on survival in 320 propensity-matched patients. This apparent difference can potentially be explained by the fact that the decision to initiate ECPR was not based on a study protocol, but was made at the discretion of the emergency physician in the latter study [28]. A meta-analysis, including eight observational studies, showed a benefit ratio of 3.2 for good neurological outcome 1 year after arrest, in favour of ECPR versus conventional resuscitation [29]. Outcome might be better in patients with signs of life during CPR, shockable rhythm and initiation of bystander basic life support. Conversely, a non-shockable rhythm or a long no-flow state is typically associated with a worse neurological outcome [30–33]. Most ECPR studies have therefore used those so-called favourable and non-favourable indicators as inclusion and exclusion criteria.

Results from several randomised clinical trials addressing the efficacy and effectiveness of ECPR for OHCA trials have recently become available (Tab. 3). The ARREST trial was the first clinical trial randomising OHCA patients to ECPR or standard advanced cardiac life support (ACLS). After enrolment of 30 patients, the trial was prematurely stopped because the pre-defined criteria for the superiority of ECPR over conventional therapy had been reached. The survival to discharge rate was 43% in the ECPR group versus only 7% in the standard CPR group, while survival with favourable neurological outcome at 6 months was 43% versus 0%, respectively [34]. While these results seemed extremely promising, the small number of patients included and the single-centre design complicated the generalisability of the study results. The second study to be published was the Prague OHCA study, which investigated a bundle of interventions, including swift transport with mechanical CPR, ECPR in the catheterisation laboratory in patients without ROSC, and coronary or pulmonary angiography [35]. This was compared to a control group with normal care without these interventions. The study randomised 256 patients and found no difference in survival with minimal or no neurological impairment at 180 days, the primary outcome, but a significant difference in the secondary outcome, i.e. favourable neurological outcome after ECPR 30 days after arrest. In a per-protocol analysis of the same study, the ECPR group had a significantly higher proportion of favourable neurological outcomes (21% vs 1.2%) [36].

**Table 3:** A summary of randomised clinical trials evaluating the effects of extracorporeal cardiopulmonary resuscitation (*ECPR*)

Study	Age, median (range/IQR/SD) years ECPR vs ACLS	Male gender (%) ECPR vs ACLS	Shockable rhythm (%) ECPR vs ACLS	Time from collapse to ECMO flow in ECPR group (IQR), min	Population	Intervention	Control	Patients	Primary outcome definition	Methodology	Pre-defined criteria for superiority	Outcome intervention group	Outcome control group	*p*-value or 95% CI
ARREST	59 (43–73) vs 58 (36–71)	14 (93) vs 11 (73)	15 (100) vs 15 (100)	59	OHCA with VF/VT, refractory to 3 shocks	Early ECPR in cath lab	Standard ACLS resuscitation on site and during intra-arrest transport	15 ECPR vs 15 regular care	Survival to hospital discharge	Bayesian	Posterior probability threshold of 98.6%	43.0%	7.0%	Absolute risk difference 36%, 95% CI 3.7–59.2%
Prague	59 (48–66) vs 57 (47–65)	102 (82) vs 110 (83)	72 (58) vs 84 (64)	61 (55–70)	Witnessed OHCA of presumed cardiac origin	Mechanical compression, swift intra-arrest transport and ED ECPR	Standard ACLS resuscitation on site	124 ECPR vs 132 regular care	Survival with minimal or no neurological impairment at 180 days	Frequentist	15% increase of primary outcome in intervention group	31.5%	22.0%	*p* = 0.09
INCEPTION	54 (± 12) vs 57 (± 10)	63 (90) vs 57 (89)	69 (99) vs 63 (98)	74 (63–87)	OHCA with initial VF/VT not reacting to 15 min of ACLS	Early ECPR according to local protocol	Standard ACLS resuscitation on site and during intra-arrest transport	70 ECPR vs 64 regular care	Survival with CPC 1–2 at 30 days	Frequentist	22% increase of primary outcome in intervention group	20.0%	16.0%	*p* = 0.518

*IQR* interquartile range, *SD* standard deviation, *ACLS* advanced cardiovascular life support, *CI* confidence interval, *OHCA* out-of-hospital cardiac arrest, *ED* emergency department, *VF/VT* ventricular fibrillation/ventricular tachycardia, *CPC* cerebral performance category

The INCEPTION trial was a multicentre trial performed in 10 Dutch cardiosurgical centres that randomised 160 patients with a presumed refractory OHCA due to ventricular arrhythmias to undergo either ECPR or conventional ACLS [37]. The arrest was considered refractory when it persisted despite 15 min of ACLS. All patients were transported to the hospital and randomisation occurred pre-hospital to provide the receiving hospitals with time to prepare for an ECPR procedure. Twenty-six patients were excluded between randomisation and hospital admission due to contra-indications that were unknown at the time of randomisation. Eventually, 70 patients were allocated to ECPR, of which 52 (74%) actually underwent ECPR. Sixty-four patients were assigned to conventional ACLS, of which 3 patients were crossed over to undergo ECPR. The intention-to-treat analysis showed no benefit of ECPR with regard to the primary endpoint: 30-day survival with good neurological function (odds ratio [OR] 1.4 [0.5–3.5], *p* = 0.518). Hospital survival in the patients that were randomised to ECPR and that actually underwent ECPR was 20%.

The large difference between the outcomes of the INCEPTION trial and the Prague OHCA study, but particularly the ARREST trial, can probably be ascribed to the multicentre versus single-centre approach. In the INCEPTION trial, the case load per site was substantially lower than in the ARREST trial and the Prague OHCA study, and all sites were allowed to apply a local ECPR protocol based upon local extracorporeal life support logistics and resuscitation logistics. This seems to be reflected in a longer total and in-hospital low-flow time. However, it is also noteworthy that the proportion of patients regaining ROSC despite an initial diagnosis of refractory cardiac arrest was much higher in the INCEPTION trial and in the Prague OHCA study than in the ARREST trial, which may suggest that the patients that actually stall in their cardiac arrest despite good ACLS have more severe underlying disease, as outlined above.

**Fig. 2:**
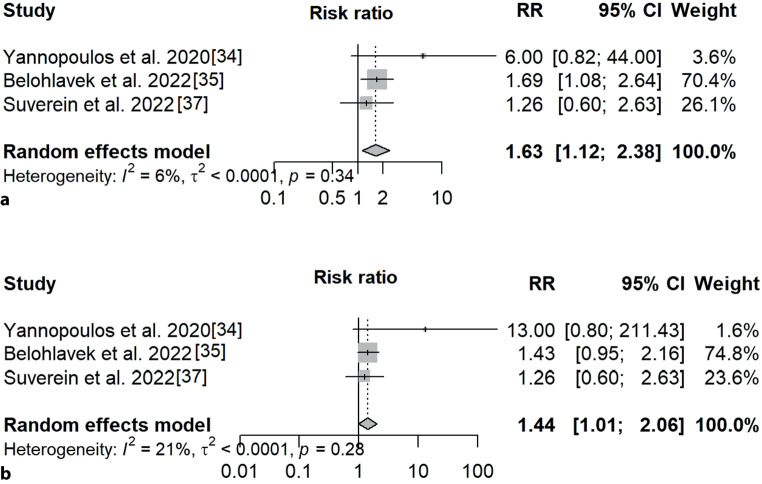
Forest plot of 1‑month (**a**) and 6‑month (**b**) outcomes

Two of the three randomised clinical trials were terminated prematurely on the basis of advice from the Data and Safety Monitoring Board. In the ARREST trial, this was due to the fact that the accrued probability for the superiority of ECMO exceeded a predetermined stopping criterion. In contrast, the reason for termination of the Prague OHCA study was a predefined stopping rule for futility. Pooling the data from the three randomised trials shows that ECPR is associated with improved outcomes over standard care at 1 month (risk ratio [RR] 1.63, 95% confidence interval [CI] 1.12–2.38) and 6 months (RR 1.44, 95% CI 1.01–2.06), while inter-study heterogeneity was low (*I*^2^ 6 and 21%, respectively) (Fig. 2).

No randomised clinical trials exist for the situation where in-hospital cardiac arrest occurs. Multiple single-centre observational studies have shown favourable results using ECPR in a selected patient category. Two independent propensity-matched observational studies showed that survival with good neurological outcome was twofold higher with ECPR as compared to conventional CPR [38, 39]. A meta-analysis in patients with cardiogenic shock or patients with refractory cardiac arrest after acute myocardial infarction showed that ECPR was associated with an absolute increase of 13% in survival at 30 days in comparison to conventional therapy [40].

## Shortening the time to ECMO

The majority of ECPR programmes require transportation of a patient to a hospital prior to cannulation. The absence of a readily available ECPR team often results in an important delay, which might explain the contradictory results. Therefore, all involved in thoracic intensive care at our centre (Erasmus MC, Rotterdam) underwent a thorough cannulation training programme to provide 24/7 ECPR coverage to reduce the waiting time in the emergency room. At the start of the INCEPTION trial this service was available at the Erasmus MC and resulted in a significantly reduced time from collapse to ECMO flow (60 min vs 75 min). This is known to be an important factor for the improved survival rate in the ECPR group compared to the control group.

Prolonged intra-arrest transport is associated with worse outcomes [41, 42]. This is likely due to an extended low-flow time and the negative impact of two transfers (to and from the ambulance trolley) on the quality of chest compressions en route. This could particularly be true in the absence of automated chest compression devices [41, 42]. These considerations led to the hypothesis that ECPR might be more effective if implemented in the pre-hospital setting. Current evidence on pre-hospital ECMO is very limited, and most publications report on small case series. The largest pre-hospital ECPR study for OHCA was conducted in Paris and its surroundings and was based on retrospective data acquired between 2011 and 2018 [43]. Of the almost 13,000 patients with OHCA, 525 and 136 patients received ECPR in an in-hospital and out-of-hospital setting, respectively. Although ECPR in general (both in- and pre-hospital implementation) was not associated with a survival benefit, application of ECPR specifically in the pre-hospital setting was significantly associated with improved survival (OR 2.9; *p* = 0.002). A propensity-matched cohort study comparing two time periods showed a higher survival rate (29% vs. 8%, *p* < 0.001) in the time frame in which ECPR was predominantly deployed in the pre-hospital setting versus the second time frame when ECPR was mainly used only in the hospital setting [30].

**Fig. 3:**
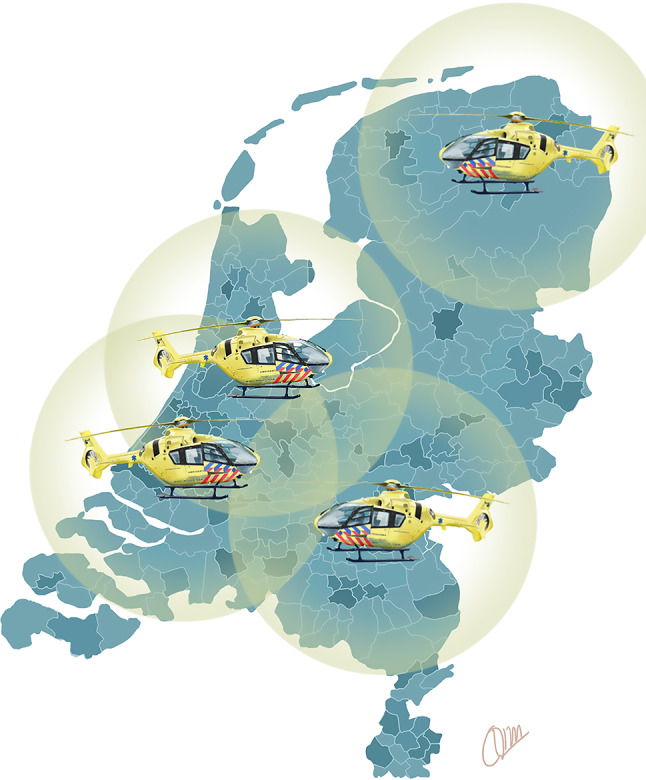
Extracorporeal cardiopulmonary resuscitation performed by helicopter emergency medical services: geographical area covered in the Netherlands. The locations of the four helicopter teams in the Netherlands are indicated. The *circles* indicate the geographical area which can be reached within 20 min of flight time. Drawn by C.L. Meuwese

Evidence from these observational studies has led to the design and initiation of the ON-SCENE trial (www.onscenetrial.com). This nationwide pre-hospital ECPR programme started in the Netherlands in January 2022. Helicopter emergency medical services (HEMS) will initiate pre-hospital ECPR in patients aged between 18 and 50 years with sustained witnessed OHCA. The HEMS system, covering a wide geographical area (Fig. 3), aims at a low-flow time of up to 40 min, thereby potentially improving the rate of favourable neurological outcomes. As such, the ON-SCENE trial constitutes the first nationwide ECPR study in the pre-hospital setting using the HEMS and focussing on young (< 50 years) patients, with a large potential improvement in survival and quality of life.

## Societal implications of ECPR

The initiation and maintenance of an ECPR programme bring forth significant practical and economic consequences. On the basis of observational studies, the average costs for an ECPR patient were calculated to be approximately € 52,000 [44]. More than half of these costs were incurred for hospital nursing days, while the ECPR procedure itself accounted for approximately 11% of the total cost. The total costs for ECPR would translate into about € 16,890 per gained QUALY [45]. This amount would be far less than the willingness-to-pay thresholds of € 50,000–100,000 in western countries.

A robust cost-effectiveness analysis on the basis of randomised data has not yet been published. The pre-planned cost-effectiveness analysis of the INCEPTION trial is therefore anxiously awaited. It must, however, be noted that, as with every novel therapy, initial costs are higher than after routine implementation. As time goes by and a therapy becomes conventional, costs will likely decline. Results from the pre-planned cost-effectiveness analysis of the INCEPTION study could provide a fundament for a societal debate on the proportionality of ECPR in the Netherlands. The ongoing increase in healthcare expenditure and the expected shortage of health personal require not only an indication of cost-effectiveness, but also thorough evaluation of cost-efficiency.

## Conclusion

Although overall survival rates for OHCA patients have improved over the last two decades, virtually no progress has been made for a subset of patients with refractory cardiac arrest, for whom survival remains very poor. ECPR was recommended for these patients in order to achieve life-saving effects. Although two of the three conducted randomised clinical trials failed to show a significant effect on outcomes, a pooled analysis of these results suggests a more favourable effect of ECPR as compared with conventional care. However, before ECPR can be considered standard of care, significant progress must be made. This could potentially be accomplished by shortening the low-flow time and more stringent selection of patients. To this extent, a unique nationwide study (ON-SCENE trial) deploying ECPR in the pre-hospital setting in young patients (< 50 years) with favourable resuscitation characteristics is currently underway and will provide data that will assist the societal discussion on the role of ECPR in the Netherlands.
